# Unilateral cleft lip and palate patients present cranial base modifications: a cross-sectional study

**DOI:** 10.1590/1807-3107bor-2025.vol39.004

**Published:** 2025-01-13

**Authors:** Eduardo Murad VILLORIA, Bernardo Quiroga SOUKI, Marina Araújo Leite ASSIS, Dauro Douglas OLIVEIRA, Thaís de Lima AZEREDO, Rodrigo Villamarim SOARES

**Affiliations:** (a) Universidade Federal do Rio de Janeiro – UFRJ, School of Dentistry, Professional Master’s Program in Dental Clinic, Rio de Janeiro, RJ, Brazil.; (b) Pontifícia Universidade Católica de Minas Gerais – PUC Minas, School of Dentistry,Graduate Program in Orthodontics, Belo Horizonte, MG, Brazil.; (c) Pontifícia Universidade Católica de Minas Gerais – PUC Minas, School of Dentistry, Graduate Program in Dentistry, Belo Horizonte, MG, Brazil.

**Keywords:** Cleft Lip, Cleft Palate, Skull Base, Cone-Beam Computed Tomography

## Abstract

The aim of this cross-sectional study was to perform a three-dimensional (3D) assessment of the cranial base of patients with unilateral cleft lip and palate (UCLP). Cone-beam computed tomography (CBCT) scans of 52 UCLP patients (21 females and 31 males; mean age, 10.0 ± 2.12 years) were compared with the scans of 72 individuals (24 females and 48 males; mean age, 11.0 ± 2.11 years) without CLP, matched by gender and age (control group, CG). The 3D Euclidean distances of anterior cranial base (N-S), posterior cranial base (S-Ba), total cranial base (N-Ba) lengths, cranial base width (Po-Po), as well as the cranial base flexure (NSBa), were measured using open-source software ITK-SNAP and 3D Slicer. Statistical analyses were carried out with the Student’s t-test at a significance level of 5%. UCLP demonstrated shorter 3D distances than CG in the N-S, S-Ba, and N-Ba cranial base lengths (p < 0.001). In comparison with female CG, female UCLP had a smaller cranial base flexure (NSBa; p = 0.020). No statistically significant differences between UCLP and CG were found for the cranial base width (Po-Po). UCLP patients presented distinct morphological cranial base characteristics in comparison with CG. These results indicate that morphological and positioning changes in the maxillary bones are not solely attributable to the cleft and/or surgical procedures.

## Introduction

Cleft lip and palate (CLP) is a common congenital craniofacial anomaly, affecting approximately 700 live births.^1^ It is caused by failures in the fusion and growth of the facial processes during embryonic development, between the fourth and twelfth weeks of gestation, and has a multifactorial etiology involving genetic and environmental factors.^2^ Although CLPs do not constitute a threat to life, they can have a significant impact on an individual’s quality of life.^3^ Changes in the facial growth pattern, primarily affecting development of the maxilla, and middle third of the face, appear on a regular basis, and are the result of both the abnormality and surgeries performed on the infants since birth.^4,5^

The cranial base, which connects the bones of the skull to bones of the face, plays a crucial role in facial development and growth.^5^ The size and shape of the cranial base affect face length, maxillary and mandibular spatial position, and size.^6^ The results of linear (N-S, S-Ba, and N-Ba) and angular (NSBa) measurements of the cranial base in CLP patients remain controversial.^7-12^ In this regard, an inaccurate assessment of the dimensions of this anatomical structure can affect the treatment planning for orthodontics or maxillofacial surgery, compromising esthetics and stability of the clinical outcomes.^13^

A significant amount of research on the cranial base morphology in patients with CLP has been conducted using radiographic assessments, and there has been no consensus.^3,8,9,12^ Cone-beam computed tomography (CBCT) offers image assessment in its true dimension (1:1), in three planes (axial, coronal, and sagittal), with a lower radiation dose than multi-slice computed tomography.^14-17^ Moreover, tools created for three-dimensional analysis of anatomical structures enables in-depth understanding of the maxillomandibular morphology in patients with facial anomalies and pathologies.^10,18-20^

To the best of our knowledge, only two studies^10,13^ have evaluated the cranial base morphology of patients with CLP using CBCT. Since some methodological limitations, such as a small sample size, were reported^10^, the purpose of the present study was to perform a three-dimensional (3D) assessment of the cranial base of patients with unilateral cleft lip and palate (UCLP) using CBCT scans.

## Methods

### Study sample

This retrospective cross-sectional investigation was approved by the institutional review board of the Pontifical Catholic University of Minas Gerais (IRB approval number: 61531416.8.0000.5137) and conducted in accordance with the tenets of the Declaration of Helsinki.

In accordance with STROBE^21^ guidelines, 126 CBCT scans and orthodontic records of 126 patients treated at the Graduate Program in Orthodontics of Pontifical Catholic University of Minas Gerais between January 2010 and December 2018 were included in this investigation.

The inclusion criteria for UCLP group were: 1) CBCT scans acquired before the treatment; 2) the presence of complete UCLP; 3) history of primary surgical interventions only; and 4) absence of orthodontic treatment. The exclusion criteria were: 1) patients with CLP other than the unilateral, 2) patients with any other craniofacial anomaly; 3) CBCT scans with technical errors in their acquisition; and 4) patients with history of secondary surgical procedures (grafts, for example).

CBCT scans were acquired in the same equipment (i-CAT Classic, Imaging Sciences International, Hatfield, Pa, USA) with a 21 cm x 17 cm field of view, 120 Kv, 8 mA, 40 seconds of exposure time and an isotropic voxel of 0.3 mm, following the university imaging center protocol.

### CBCT processing and measurement method

**Figure 1:**
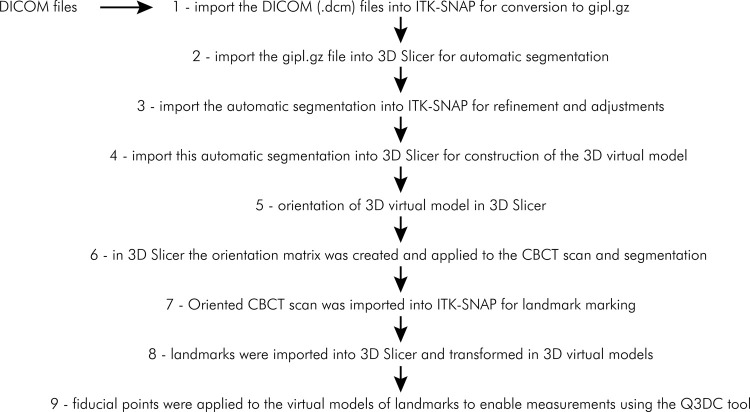
Flowchart of the CBCT scans processing method for three-dimensional measurements.

For processing CBCT images and 3D virtual models, the ITK-SNAP version 2.2 (open-source software, www.itksnap.org) and 3D Slicer version 4.5.0 (open-source software, www.slicer.org) were used as described below^22^ and illustrated in the flowchart (Figure 1).

Initially, DICOM files (.dcm) were imported into ITK-SNAP for conversion into gipl.gz files. Then, the gipl.gz file, referring to the CBCT scan, was imported into the 3D Slicer to perform automatic segmentation using the “intensity segmenter” module. After automatic segmentation in the 3D Slicer, the file was imported into ITK-SNAP to refine and adjust segmentation for removing structures that would not be important for this study.

The gipl.gz file referring to the refined segmentation in ITK-SNAP was imported into the 3D Slicer for constructing the 3D virtual model using “Model Maker” module. Then, using the “transforms” module, 3D virtual models were oriented in the Cartesian plane, as previously described.^22,23^ Subsequently, using the “apply matrix” module, the orientation matrix was created and applied to the CBCT scan and segmentation.

**Figure 2:**
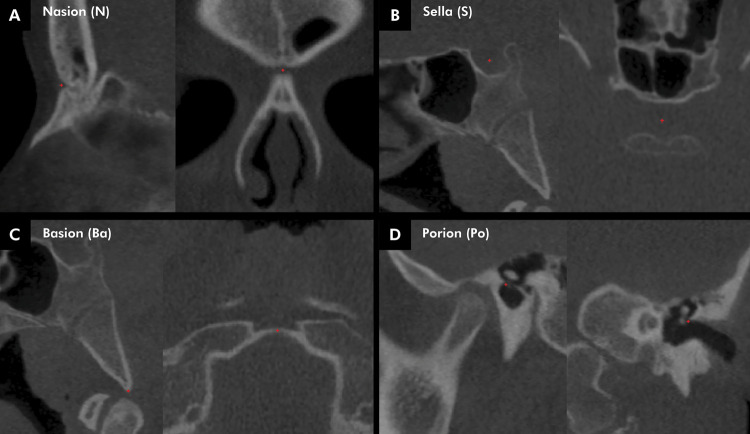
Landmarks in red color applied to multiplanar CBCT slices. Nasion (A), sella (B), basion (C) and porion (D) cephalometric points.

The oriented CBCT scan in the gipl.gz file was again imported into ITK-SNAP to identify and apply landmarks. The landmarks, in sphere format and size 3 (3 voxels = 0.9 mm in diameter), were marked at cephalometric points N, S, Ba and Po on the right and left sides (Figure 2). The points, located in multiplanar planes (axial, coronal and sagittal), and all landmark markings were performed by a unique and experienced Oral Radiology, trained, and experienced in the use of these software (E.M.V.).

**Figure 3:**
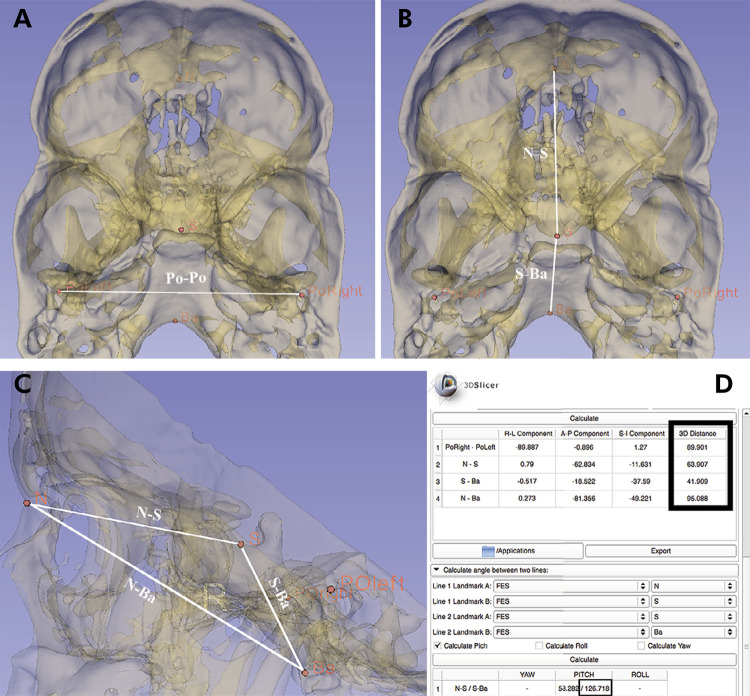
Three-dimensional virtual model of the cranial base in 3D slicer software. Top (A and B) and lateral (C) views demonstrating the fiducial points applied on the landmarks PoRight, PoLeft, N, S and Ba; 3D Euclidean distances between Po-Po, N-S, S-Ba, N-Ba and NSBa angle in the Q3DC module (D).

The landmarks were saved in gipl.gz and imported into the 3D Slicer. A three-dimensional virtual model for each landmark was created and “fiducial points” were applied on the virtual models of these landmarks, allowing measurement of NSBa angle and N-S, S-Ba, N-Ba and Po-Po distances by using the “Q3DC” module of 3D Slicer itself (Figure 3). As described previously,^22,24^ the NSBa angle, in sagittal view, was measured as pitch rotation. Linear measurements were measured in the 3D Euclidean distance.

In order to verify intra-observer variability and reproducibility, ten CBCT scans were randomly selected, and marking of landmarks and measurement of linear distances were performed twice, with an interval of 15 days. The intraclass correlation coefficient (ICC) ranged from 0.80 to 0.99, which demonstrated a high level of reliability.

### Statistical analysis

The SPSS software version 21.0 (SPSS Inc., Chicago, Illinois, USA) was used. The chi-square test was performed to verify homogeneity of the categorical and independent variable (sex). Certification of the assumptions of normal distribution and same variance was carried out in the continuous and dependent variables of the study, using the Kolmogorov-Smirnov and Levene tests, respectively. The Student’s t-test was performed considering a significance level of 5%.

## Results

After an initial analysis of the total sample, 2 CBCT scans were excluded because they had a post-foramen cleft lip and palate. A total of 52 CBCT scans from patients with complete UCLP (21 females and 31 males) patients, with a mean age of 10.0 ± 2.12 years, were included. The control group (CG) consisted of 72 non-cleft patients or other craniofacial anomaly (24 females and 48 males) with a mean age of 11.0 ± 2.11 years. Patient demographics are shown in Table 1, and Table 2 demonstrates homogeneity of the sex variable (p = 0.420).

**Table 1:** Patient demographics.

Variables	UCLP	CG
	n = 52 (42%)	n = 72 (58%)
Age (Mean ± Standard deviation)	10.0 (2.12)	11.0 (2.11)
Sex n (%)		
Male	31 (59.6%)	48 (66.6%)
Female	21 (40.4%)	24 (33.4%)

UCLP: individuals with unilateral cleft lip and palate; CG: individuals without cleft lip and palate.

**Table 2:** Assessment of the sex variable homogeneity

Group	Sex		p-value
	Male	Female	
UCLP	31	21	0.420*
CG	48	24	

UCLP: individuals with unilateral cleft lip and palate; CG: individuals without cleft lip and palate; *Chi-square test; ^**^Statistically significant difference (p < 0.05).

Statistically significant differences were found in the 3D Euclidean distance (length) of the cranial base of UCLP in comparison with CG, as shown in Table 3. The anterior (N-S), posterior (S-Ba) and total (N-Ba) cranial base lengths of UCLP were shorter than CG (p < 0.001). Relative to cranial base width (Po-Po), and cranial base flexure (NSBa), no statistically significant differences between UCLP and CG were found.

**Table 3:** Angular and 3D Euclidean linear measurements of the UCLP and CG groups.

Measure	Component	Group UCLP (52) / CG (72)	Mean	SD	p-value
NSBa	Pitch ^*^	UCLP	130.52	6.19	0.206
		CG	131.88	5.65	
N-S	3D	UCLP	62.31	2.86	< 0.001^**^
		CG	65.75	3.89	
S-Ba	3D	UCLP	40.20	3.08	< 0.001^**^
		CG	43.07	3.07	
N-Ba	3D	UCLP	92.81	6.28	< 0.001^**^
		CG	99.67	5.68	
Po-Po	3D	UCLP	87.36	5.18	0.098
		CG	88.78	4.27	

*Pitch: angular measurements following the rotational direction of the lines up and down, in anteroposterior direction. **Statistically significant difference (p < 0.05).

Comparison of angular and linear measurements between male and female UCLP and CG is presented in Table 4. The N-S, S-Ba and N-Ba of UCLP of both sexes were smaller than CG (p < 0.05). Relative to NSBa, only female UCLP had a significantly small measurement in comparison with CG (p < 0.05). No differences were found between UCLP and CG relative to the Po-Po measurement, considering the sex variable.

**Table 4:** Angular and 3D Euclidean linear measurements relative to males and females

Variable	Component	Group	Sex					
			Male			Female		
			UCLP (31) / CG (48)			UCLP (21) / CG (24)		
Measure			Mean	SD	p-value	Mean	SD	p-value
NSBa	Pitch*	UCLP	131.78	6.51	0.777	128.67	5.29	0.020**
		CG	131.39	5.37		132.87	6.19	
N-S	3D	UCLP	63.02	2.66	< 0.001**	61.27	2.89	0.005**
		CG	66.68	3.98		63.90	3.00	
S-Ba	3D	UCLP	40.83	2.67	< 0.001**	39.26	3.47	0.002**
		CG	43.55	3.27		42.10	2.38	
N-Ba	3D	UCLP	94.02	7.01	< 0.001**	91.02	4.61	< 0.001**
		CG	100.80	6.00		97.40	4.23	
Po-Po	3D	UCLP	88.33	5.29	0.187	85.93	4.78	0.471
		CG	89.70	3.85		86.95	4.55	

*Pitch: angular measurements following the rotational direction of the lines up and down, in anteroposterior direction. **Statistically significant difference (p < 0.05).

## Discussion

Patients with CLP frequently present maxillary constriction and are prone to show modifications in the mandibular position and cranial base morphology.^7,12^ However, relative to some linear measurements (N-S, S-Ba, and N-Ba), and the cranial base flexure (NSBa), evidence in the literature is still controversial.^7-12^ Small sample sizes, non-homogeneity of independent variables, and a lack of standardizing imaging acquisition and/or analysis (cephalograms versus CBCT) can explain this divergence.^7-12^

Using CBCT, only two investigations^10,13^ have examined the cranial base morphology in CLP patients. One performed a 3D evaluation using multiplanar reconstructions and 3D virtual models,^10^ while the other performed a 2D evaluation with use of a sagittal CBCT view.^13^ Contrary to the findings of the present study, one of the previous investigations^10^ detected no statistically significant difference between S-N, S-Ba, N-Ba and NSBa. However, the limited sample size of the previous study (14 patients with UCLP) may have led to a type II error. Moreover, the landmarks were marked directly on the 3D model as opposed to the CBCT scan. This limitation was presented by the authors, who suggested conducting additional three-dimensional studies with larger sample sizes to evaluate the cranial base morphology in CLP patients. The difference between the previously reported results^13^ and present study results can be partially attributed to the CLP group consisting of patients aged 18 to 70 years (mean age, 32.1 years), small sample size (14 female and 6 male patients), absence of an analysis of homogeneity for this variable, and CLP type.

Similar to findings from a recent systematic review^25^, UCLP patients in the present study exhibited a smaller 3D distance in N-S, S-Ba and N-Ba, indicating significantly less development of the anterior, posterior, and total length of this anatomical structure when compared with CG patients. However, the Po-Po distance failed to indicate a statistically significant difference in the cranial base width between UCLP and CG patients.

In the aforementioned CBCT studies^10,13^as well as previous 2D studies^8,9,12^, NSBa did not demonstrate a statistically significant difference between UCLP and CG patients. In this regard, a recent meta-analysis found that patients with CLP had a more obtuse cranial base angle than those without CLP. However, the authors reported a high degree of heterogeneity between studies due to differences in age, gender, ethnicity, cleft severity, and surgical procedures.^25^

Male and female UCLP patients had significantly smaller 3D Euclidean distances in N-S, S-Ba and N-Ba compared with CG, as determined by analysis of the sex variable conducted in the present study. Only female UCLP patients had a smaller NSBa, indicating that female UCLP patients were genetically predisposed to have a smaller cranial base angle, which would be likely to exacerbate class III. A unique study published in 1982 found no gender-specific variations in the cranium base angle in CLP patients^26^, and no other studies have evaluated the morphology of the cranial base in males and females in recent years. Therefore, the potential differences in craniofacial morphology between male and female cleft populations have not been thoroughly investigated.^25^ The association between abnormal cranial base morphology and cleft palate can be explained anatomically and functionally.^27^ From an anatomical perspective, the cranial base is a structure that attaches the neurocranium to the facial skeleton. Consequently, cranial base development and growth may interact with neurocranial and facial skeletal development. Moreover, since the sphenooccipital synchondrosis represents remnants of the early chondrocranium, it is feasible that a congenital alteration, abnormal growth, or late maturation of the cartilage tissue could affect the morphology of the cranial base.^28^Functionally, factors such as the extended head posture induced by diminished size of the airways can also interfere with the postnatal growth pattern of the cranial base in individuals with CLP.^29^

Furthermore, anteroposterior development of the cranial base is closely related to the positioning of the maxilla and mandible, which contributes to the degree of facial prognathism.^30^ The shortened anterior cranial base length contributes to a shallower midface, resulting in a more posteriorly positioned maxilla relative to the mandible.^31^ Moreover, the more acute cranial base angle may favor a more anterior positioning of the mandible and, consequently, development of Class III malocclusion^8^, which is commonly observed in CLP patients. The lower anteroposterior development of the cranial base and lower angle of the cranial base observed in female UCLP patients suggest that this condition may contribute to maxillary retrognathism and mandibular prognathism commonly observed in UCLP patients. Findings that confirm previous descriptions that maxillofacial development of CLP patients is significantly influenced by non-surgical factors.^9^

Due to the retrospective cross-sectional design with a convenience sample, it was not possible to classify UCLP patients according to Angle’s system. As there were no differences in the distribution of types of malocclusion and sex between the two groups, this limitation should have no bearing on the results observed. In addition, we recommend future studies that can organize the groups according to Angle’s classification and increase the sample size based on the calculation of the sample size.

## Conclusions

Young patients of both sexes, with complete UCLP, had a shorter anteroposterior cranial base length (N-S, S-Ba, and N-S) than the CG group. No differences in cranial base width (Po-Po) were observed. The cranial base angle (NSBa) was smaller only in female UCLP patients. UCLP patients have morphological changes at the base of the cranium. Therefore, morphological and positioning changes in the maxillary bones are not exclusively attributable to the cleft and/or to surgical procedures.
